# How supervisor trust affects early residents’ learning and patient care: A qualitative study

**DOI:** 10.1007/s40037-021-00674-9

**Published:** 2021-07-23

**Authors:** Brian C. Gin, Stephanie Tsoi, Leslie Sheu, Karen E. Hauer

**Affiliations:** 1 Department of Pediatrics, University of California—San Francisco, San Francisco, CA USA; 2 Department of Medicine, University of California—San Francisco, San Francisco, CA USA

**Keywords:** Entrustment, Trust, Autonomy, Supervision, Trainee, Residency

## Abstract

**Introduction:**

Trust between supervisors and trainees mediates trainee participation and learning. A resident (postgraduate) trainee’s understanding of their supervisor’s trust can affect their perceptions of their patient care responsibilities, opportunities for learning, and overall growth as physicians. While the supervisor perspective of trust has been well studied, less is known about how resident trainees recognize supervisor trust and how it affects them.

**Methods:**

In this qualitative study, 21 pediatric residents were interviewed at a single institution. Questions addressed their experiences during their first post-graduate year (PGY-1) on inpatient wards. Each interviewee was asked to describe three different patient care scenarios in which they perceived optimal, under-, and over-trust from their resident supervisor. Data were analyzed using thematic analysis.

**Results:**

Residents recognized and interpreted their supervisor’s trust through four factors: supervisor, task, relationship, and context. Optimal trust was associated with supervision balancing supervisor availability and resident independence, tasks affording participation in decision-making, trusting relationships with supervisors, and a workplace fostering appropriate autonomy and team inclusivity. The effects of supervisor trust on residents fell into three themes: learning experiences, attitudes and self-confidence, and identities and roles. Optimal trust supported learning via tailored guidance, confidence and lessened vulnerability, and a sense of patient ownership and team belonging.

**Discussion:**

Understanding how trainees recognize supervisor trust can enhance interventions for improving the dialogue of trust between supervisors and trainees. It is important for supervisors to be cognizant of their trainees’ interpretations of trust because it affects how trainees understand their patient care roles, perceive autonomy, and approach learning.

**Supplementary Information:**

The online version of this article (10.1007/s40037-021-00674-9) contains supplementary material, which is available to authorized users.

## Introduction

A growing body of literature identifies supervisor trust as a key component of trainees’ experiential learning in the health professions. Trust can be considered as the affective and cognitive factors that influence a trustor’s decision to delegate a responsibility to a trustee [1], whereas the term *entrustment* refers to a clinical supervisor’s decision to transfer responsibility for a task to a trainee [2]. The trust a supervisor has in a trainee mediates opportunities for trainees to participate progressively and assume clinical independence [3]. Because effective trust requires both entrustment from the supervisor and engagement by the trainee, it is important to understand how trainees recognize and respond to their supervisors’ trust.

Initial studies of entrustment focused on supervisors’ perspectives [4–7], but more recently, the discussion around trust has shifted to trainees’ perspectives [8–10]. For medical students, supervisor trust supports the scaffolding that generates opportunities for clinical learning and participation [11]. Studying resident trainees’ perspectives of entrustment revealed actionable ways for trainees to earn trust from their supervising attending physician [12]. A study of how residents manage clinical uncertainty revealed a role for trusting relationships in promoting residents’ autonomy and reciprocity of trust back to their supervisors [13]. This notion of mutual trust is thought to arise from early affective impressions, followed later by cognitive judgements from both supervisor and trainee viewpoints [14].

Less is known about how trainees recognize supervisor trust. Assessing trainees’ understanding of their supervisors’ trust is important for supervisors to know whether they are communicating trust effectively, and whether trainees are interpreting the intended degree of trust accurately. In addition, trainees’ recognition and interpretation of supervisor trust may affect how they understand their roles in patient care, perceive autonomy, approach learning, and ultimately grow into independent physicians. How residents interpret and respond to supervisor trust is particularly important in the first year of postgraduate training (PGY-1) because trainees’ early perspectives have the potential to affect their overall residency experience and development as physicians [15]. During this year, trainees gain increasing patient care responsibilities and, for the first time, are granted autonomy to act without direct supervision as physicians.

Several studies have corroborated five factors that physician supervisors consider when entrusting resident trainees: supervisor, trainee, relationship, task, and context [4–7]. While prior work has not directly applied these factors to the trainee perspective, similar themes can be seen in studies on trainee perspectives of feedback and autonomy [16, 17]. Utilizing these factors as a sensitizing framework [18], this study aimed to (1) understand how residents recognize supervisor trust and (2) explore the effects of residents’ perceptions of supervisor trust on their learning and patient care. Clarifying the trainee perspective of trust during residency can improve how supervisors and trainees work together to deliver patient care and facilitate trainee learning and progression.

## Methods

### Study design

We conducted a qualitative interview-based study to investigate perceptions of pediatric residents during PGY‑1.

### Participants and setting

PGY‑1 and 2 residents in the 3‑year pediatric residency at the University of California, San Francisco (UCSF) in the United States were eligible to participate after completing at least one month of inpatient pediatrics wards. During inpatient wards rotations, PGY‑1 residents at UCSF are primarily supervised by “senior” residents (PGY-3). We invited all 60 PGY‑1 and PGY‑2 residents from the 2014–2015 academic year, and all 30 PGY‑1 residents from the 2015–2016 academic year for individual interviews. Interviews occurred from February to October 2015 and took place during the first half of the academic year for PGY-1 participants, and after completion of the PGY-1 year for PGY‑2 residents. The UCSF institutional review board deemed this study exempt (UCSF IRB File no. 14-15087).

### Interview guide and data collection

Three authors (BG, LS, KEH) developed a semi-structured interview guide (see Appendix in the Electronic Supplementary Material [ESM]) based on the critical incident technique—a method chosen to identify salient experiences related to a broad range of trust [19]. We asked interviewees to recall patient care scenarios they experienced as PGY‑1 residents on inpatient pediatrics wards. To capture a range of levels of trust, we asked them to recall scenarios with optimal trust, under-trust, and over-trust. Authors refined the interview guide for clarity through three pilot interviews, which were included in the final data set. A single author (BG) performed 21 interviews in person and by telephone, which were recorded, professionally transcribed, and de-identified. Interviews lasted on average 30 min. We coded transcripts concurrently with data collection and stopped interviewing when we identified no further codes and scenario descriptions became multiply redundant, signaling sufficiency [20].

### Data analysis

We performed thematic analysis using matrix exploratory methods designed to be generally applicable to all qualitative frameworks [21, 22]. We chose thematic analysis to identify patterns related to our aims across different levels of trust [23]. First, BG created short summaries of each transcript and pulled direct quotes from each transcript and generated a preliminary descriptive code list. Three authors (BG, LS, KEH) read nine initial transcripts and revised these codes iteratively [24]. BG and ST applied these codes to the remaining transcripts and constructed a partially ordered meta-matrix [25] of coded excerpts grouped vertically by the level of trust (optimal/under/over). All authors participated in iterative discussion and data review to synthesize data into larger themes, focusing on trainee perceptions of supervisor behaviors that supported trust, and on trainee perceptions of the effects of trust. We used Dedoose qualitative software for coding and analysis [26].

We considered the authors’ reflexivity in this study, with particular acknowledgement of the medical hierarchy [27]. At the time of analysis one author (ST) was a UCSF pediatric resident. Another author (BG) was a senior resident (PGY-3) at study onset and subsequently a pediatric hospitalist fellow during data collection, with no direct responsibilities in evaluating trainees. Two authors (LS, KEH) are internal medicine attendings and educational scholars. Throughout data collection and analysis, we reflected on and discussed the impact of our roles, representing both trainee and supervisor viewpoints in adult and pediatric inpatient medicine. While we believe that our discussions were enriched by our representation of diverse stakeholder groups, we also strove to make inferences apart from these perspectives.

## Results

Of 90 eligible residents, 32 volunteered to participate, and 21 were available for scheduled interviews. PGY-1 participants had been in residency training for an average of 3.5 (*SD* = 0.1) months at the time of their interview, and PGY-2 participants 19.6 (*SD* = 1.0) months. Participants included 17 (81%) women, similar to 76% in the overall program.

We grouped our results according to our two research questions: how trainees recognize supervisor trust and how trainees are affected by their perceptions of trust. We identified four codes addressing how trainees recognized supervisor trust. These codes aligned with four of the five factors from our sensitizing framework (Hauer and colleagues’ model of supervisor trust [3])—supervisor, task, relationship, and context (see ESM, Table S1). Interviewees discussed the fifth factor of this model, the trainee, not in terms of their own contributions to trust, but rather by the effects of trust on themselves. Three additional codes described the effects of trust on PGY‑1 trainees: learning experiences, attitudes and self-confidence, and identity and role (see ESM Tab. S2). Sorting interviewees’ experiences by code and trust level revealed patterns described by the themes shown in both tables.

### How trainees recognize supervisor trust

#### Supervisor

Trainees recognized different levels of trust based on the amount and type of support a supervisor provided when they faced acute or complex patients, unfamiliar cases, or difficult situations. Supervisors showed support through their preparation, teaching, availability, debriefing, and feedback. Trainees perceived *optimal trust* from their supervisors when they received what they perceived to be an appropriate amount of support. Trainees described feeling that their supervisors were invested in them when they spent time to thoroughly assess the trainee’s skills and competence, and shared expertise relevant to the trainee’s needs. Trainees recognized excessive supervisor support as *under-trust*. Trainees described behaviors such as underestimation of their capabilities by their supervisors, micromanaging, and redundancy in work. Trainees perceived *over-trust* when supervisors provided insufficient support, overestimated trainee capabilities, and were unavailable for teaching and guidance.

#### Task

Trainees recognized trust based on the type of task they were assigned to complete by their supervisors. The amount of trust perceived by the trainee was influenced by characteristics of the task, including complexity, acuity, sequencing of tasks over time, opportunity for autonomy, and associated risk to the patient. In *optimal trust* scenarios, trainees described being assigned tasks that matched their competence and appropriately promoted their growth. Trainees’ roles and responsibilities were communicated clearly from the supervisor to the trainee and promoted critical thinking and participation in patient care. Trainees recognized *under-trust* when supervisors assigned tasks in ways that restricted their learning and growth, limiting their roles and responsibilities. This included exclusion from decision-making processes, and assignment of low acuity, quantity, and/or complexity of tasks based on the trainee’s self-assessed competence. Trainees recognized *over-trust* when tasks exceeded their self-assessed competence. They described these tasks as either overly complex, involving a patient that was too acute, or necessitating a degree of responsibility that was above their expectations.

#### Relationship

Trainees recognized trust differently depending on the trainee–supervisor working relationship. Interpersonal dynamics, values, expectations, communication, and the amount of contact between trainee and supervisor affected the relationship that grounded trainee–supervisor trust. *Optimal trust* was fostered in relationships where the trainee and supervisor had mutual trust. Mutual trust was characterized by open and honest communication, the trainee feeling valued and receiving validation as a team member, shared expectations and mental models, mutual advocacy, and bidirectional feedback. Trainees felt optimally trusted when they had multiple opportunities to interact with their supervisors and form meaningful, longitudinal relationships with them. In situations of *under-trust*, trainees described relationships that felt hierarchical and even dictatorial. Trainees recalled feeling excluded from the team, experiencing one-way communication, or having unclear roles and expectations set between the trainee and the supervisor. Trainees recognized *over-trust* when working relationships were lacking or poor. These relationships were characterized by a lack of teamwork, poor or no communication, absence of shared values or expectations, and no trust in the supervisor.

#### Context

Trainees noted how the context of the team and the workplace influenced whether they felt optimally, under-, or over-trusted. Characteristics of the workplace included opportunities for either autonomy or dependence, workload, timing (i.e., day shift vs. night shift, during rounds or sign-out, rotation switch day), and workplace culture. Trainees recognized *optimal trust* when there was a supportive workplace with ample opportunities for trainee autonomy and growth, a collaborative environment with shared workload between trainees and supervisors, and a culture that encouraged learning and independent thinking. Effective collaborations considered the trainee’s strengths while maintaining time and space for learning and autonomy. This differed from *under-trust*, where the context included barriers to participation and critical thinking, like lack of time and space for learning opportunities and autonomy, a culture of ‘getting the work done’ over learning, and timing early in the trainee–supervisor’s block together when supervisors tended to supervise more directly. In *over-trust*, trainees described barriers to asking for or receiving help, and a workload that exceeded trainee capabilities either because of high census or inadequate staffing.

### How trust affects trainees

Trainees described a range of effects stemming from their supervisors’ trust in them. We summarized those effects into three main codes: learning experiences, attitudes and self-confidence, and identity and role, to identify the nine themes shown in ESM Tab. S2.

#### Learning experiences

*Optimal trust* created a favorable personal learning experience for trainees, as supervisors appropriately guided the trainee to acquire new skills and meet learning goals. Trainees felt motivated to learn when there was encouragement to be independent, autonomous, and ask for help. Optimal trust allowed trainees to grow as clinicians: *“I felt that I learned more. I felt that I grew more … my clinical suspicions were getting more validated … Those interactions led to more opportunities to do more” *(Participant no. [PN] 1).* Under-trust* made trainees feel restricted because of limited opportunities for independence or a feeling that learning was not prioritized. The trainee could not advance as a physician because of excessive supervision, redirection without guidance, and absence of constructive feedback. *Over-trust* created variable personal learning experiences for trainees, both positive and negative. Some felt compelled to compensate for missing teaching and feedback by self-teaching or asking other co-residents, nurses, or attendings for guidance, while others used the extra trust to work through challenges they may otherwise not have tried. *“In terms of learning, there’s pros and cons. … The pro is that it really pushes you to decide something and act on it, … but the con is that you feel like you’re learning in the dark” *(PN-9).

#### Attitudes and self-confidence

*Optimal trust* positively affected trainees’ self-worth by promoting their self-confidence and allowing them to feel valued and empowered. With this level of trust, one trainee remarked *“I can remember a lot of situations where the resident just said, ‘I trust your clinical judgment. I think this is the right decision.’ That gives you a little bit more confidence” *(PN-9). Alternatively, supervisors who *under-trusted* their trainees left them feeling belittled, worthless, undermined, and with lower self-confidence. *Over-trust* had variable effects on trainees’ attitudes and self-confidence. There were examples of over-trust that led to increased confidence if the trainee was able to complete the task successfully, but if the trainee was not able to navigate the scenario, this led to doubt, fear, vulnerability, and discomfort—“*I just felt really uncomfortable because I felt like there were a lot of things that she expected me to be able to figure out on my own that I didn’t.… I always felt a little alone and vulnerable” *(PN-19).

#### Identity and role

*Optimal trust* helped trainees establish their physician identity and have a defined role on the team. Trainees felt greater patient ownership and had more opportunities for participation in both the team as well as with direct patient care. Trainees described how their role in decision-making grew with optimal trust: “*You know everyone else is thinking about the patient too, but I definitely felt like I was the one who was making the decisions” *(PN-13). When trainees felt that their supervisors *under-trusted* them, they felt powerless, marginalized, and an unimportant part of the team. Their role felt minimized due to limited ownership over patient care and decision-making. Trainees who experienced *over-trust* felt they were given a role they did not deserve, often when their supervisor was busy. Trainees described feeling lost and worried about patient safety, but at times also willing to handle the increased autonomy and responsibility.

## Discussion

**Fig. 1:**
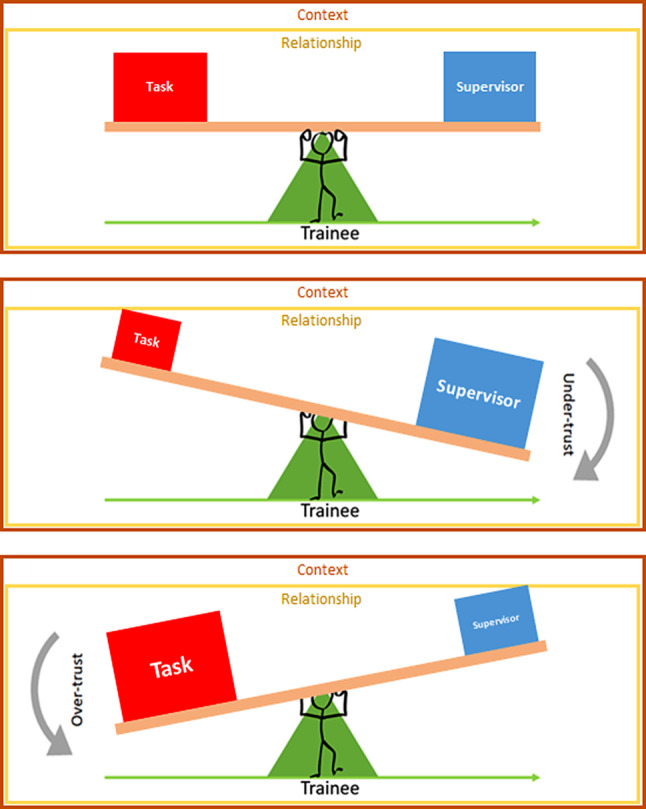
Depiction of the interplay between the five factors of trust as perceived and received by the trainee. Trainees inferred different levels of trust based on the balance between task acuity and complexity and supervisor support. This balance could be shifted depending on the context and relationship factors. This balance impacts the trainee. **a** In optimal trust, trainees perceived appropriately balanced task acuity and complexity with supervisor support; as task acuity and complexity increased so did supervisor support and vice versa. **b** In under-trust, trainees recognized low task acuity and complexity with excessive supervisor support. **c** In over-trust, trainees received high task acuity and complexity with inadequate supervisor support

This study brings new insights to trainee–supervisor trust by exploring how early resident trainees interpret and respond to different levels of their supervisors’ trust. Our results show how four of the five factors from our sensitizing framework, based on the supervisor perspective [5], can be applied to describe trainees’ recognition of supervisor trust. While our interview guide was based on these five factors, we intentionally used open-ended questions for the possibility of finding new factors; though we did not find new factors and the previously described categorization seemed to largely apply. Our findings illustrate how the *supervisor *and *task *factors directly influence perceived trust, while *relationship *and *context *contribute to the foundation for building trust. The balance of these factors creates a perceived level of trust that consequently affects the fifth factor, the trainee. We synthesize our results as a conceptual model in Fig. 1, which depicts how a trainee’s recognition of trust is dependent on the appropriate balance between the amount of supervisor support and the acuity/complexity of a task within the given working relationship and context. The perceived trust level affects the trainee’s learning experiences, attitudes and self-confidence, and identity and roles established during their first year of residency. Altogether, trainees’ responses to optimal supervisor trust seem to positively impact clinical learning and professional development.

Although the five factors were originally described to characterize supervisors’ entrustment decisions about trainees [5, 28], the applicability of these factors to the trainee perspective is not surprising given their importance to the clinical learning environment [29, 30], the appearance of similar factors in models of bidirectional trust and social interaction [1, 13, 31], and the centrality of relationship formation in theories of supervision [32–35]. This alignment may allow for the development of a common dialect encompassing both directions of trust. Adopting these five factors as a language for trust may therefore facilitate development of shared mental models between supervisors and trainees to understand, recognize, and build trust.

Our proposed model of how a trainee recognizes supervisor trust (Fig. 1) could be used in curricular interventions designed to teach trust through the balance of these five factors. Recent discussions highlight the importance of the autonomy–supervision balance in clinical learning and its outcomes on how residents form professional identities, including their sense of patient ownership, decision-making ability, and confidence [17, 36]. Sawatsky and colleagues showed that residents perceived supervisors to trust them when they were given autonomy, but also that too much autonomy could compromise learner and patient safety [17]. Our results suggest that trainees’ understanding of their supervisor’s trust comes from trainees’ beliefs about how the five factors should interplay to achieve optimal trust. Therefore, learning interventions aimed at developing awareness of the five factors of trust could support appropriate promotion along the autonomy–supervision balance [7, 37–39]. This training might come in the form of early-residency curricula specifically addressing how to recognize and negotiate trust. Later, it could adapt to residents’ roles as supervisors by discussing how to give recognizable trust, and how to address trainees’ learning needs in the context of their drive for autonomy. Developing ways for supervisors and trainees to discuss trust openly may move discussions beyond trust’s affective roots [14]—roots that may be particularly prone to unintended and unfair biases [40]. Involving trainees in explicit discussions about their entrustment may help to build trust grounded in objective decision variables based on trainee competencies rather than affective impressions [16].

Our findings highlighting the powerful impact trust can have on trainees’ attitudes and self-confidence align with self-efficacy theory [41]. Self-efficacy in the setting of medical education is described as a task-specific self-confidence that makes one feel more prepared, motivated, and capable [42]. Self-efficacy theory posits that trainees’ perceptions of their own competence depend on certain key sources, like mastery experiences and physiological or affective states [43, 44]. Interviewees positively referenced examples of these key sources when describing optimal trust, suggesting that optimal trust can increase trainees’ self-efficacy. It remains to be determined whether trust or autonomy is the primary driver of self-efficacy in this context [17]. Additionally, since recent studies have suggested a relationship between self-efficacy and well-being, it would be interesting to study whether establishing optimal trust can improve well-being through higher self-efficacy [45].

This study has limitations. We examined a single program setting in a single specialty and conducted data collection five years ago; transferability of our results to other settings or specialties may be limited. Participants volunteered to be interviewed, possibly contributing a volunteer bias and/or more passionate feelings (either positive or negative) about the topic of trust. The majority of the participants were female, which is representative of pediatrics [46], but not all specialties, and may not accurately represent the valuation of trust, although there is no study to suggest a gender dependence of trust in medical education. Additionally, participants were interviewed at different times in their training (PGY‑1 and PGY-2) and may have discussed scenarios with recall bias from subsequent experiences. Finally, representing trust as three categories (optimal, over-, and under-) may oversimplify its complexity, but our interviewees did appear to relate readily to this categorization, which enabled us to gather data on a range of experiences with trust.

Overall, this study provides a new perspective on supervisor trust from the early resident trainee viewpoint and explores the consequences of different levels of supervisor trust on trainees and their clinical growth. Adopting the five factors as a shared language between trainees and supervisors could help build trust and communicate it more effectively in the clinical learning environment. Open dialogue around finding an appropriate balance of supervisor support for a trainee’s assigned task, developing trainee–supervisor relationships, and collaborating on contextual priorities may be ways to not only improve trust, but ultimately establish mutual trust. Trainees’ perceptions of supervisor trust have important implications on their learning and patient care. Overall, our findings from the trainee perspective may help make trust more teachable, adaptable, and understandable for both trainees and their supervisors.

## Supplementary Information

Interview Guide

Table S1 Factors used by trainees to recognize different levels of supervisor trust. Four factors described how trainees recognized different levels of supervisor trust: supervisor—the amount and type of support and availability provided to trainees by their supervisors; task—the acuity, complexity, quantity, sequencing, and risk associated with clinical activities; relationship—interpersonal dynamics, values, concordance, communications, and amount of contact; and context—workplace culture and environment, systems issues, and workload. (Interviewees referred to PGY‑1 residents as “interns”, and to supervising PGY‑3 residents as “seniors”. Numbers in parentheses are participant ID numbers)

Perceived effects on the trainee from optimal, under- and over-trust. Effects were categorized into three codes which, when separated by trust level, yielded nine themes. Interviewees referred to PGY‑1 residents as “interns“, and to supervising PGY‑3 residents as “seniors.”. Numbers in parentheses are participant ID numbers
