# Supplementary material for: How supervisor trust affects early residents’ learning and patient care: A qualitative study
Source: Perspect Med Educ. 2021 Jul 23;10(6):327–33. doi: 10.1007/s40037-021-00674-9 (PMC8633204; doi:10.1007/s40037-021-00674-9)
Supplement: Supplementary file 1 — Interview Guide [file 40037_2021_674_MOESM1_ESM.docx]

**Appendix** Interview Guide

Thank you for participating in this interview. We appreciate your time and willingness to share your thoughts and experiences. Our goal is to understand, from your current or prior perspective as an intern^[[1]](#footnote-1)^, how you interacted with your supervising senior residents. Please do not use any team members or patient names or identifying information. I would like to record this interview and have it transcribed. Your name and any identifying information will be removed from the transcript and will not be used during the analysis. Is it okay with you if I record the interview?

TURN ON RECORDER

We know that interns work with varying levels of autonomy or independence in different situations, and different levels of supervision. Supervisors affect this level of autonomy by the degree of trust they place in their interns. We would like to ask you about situations where you felt the level of trust was appropriate, and cases in which you felt it was too much or too little.

First, I’d like to ask you to think back to a situation in which you felt that your resident supervisor trusted you too little.

What was the situation? Could you describe the clinical setting? How much supervision did you receive from your senior resident?

probe if not already mentioned:

*Acuity of the patient*

*Your familiarity w/ patient, length of time in care*

*Time of day (was it a nightshift/weekend?)*

*Overall ward census*

*Team “mood”*

What specifically made you feel that your resident didn’t trust you enough?

Did you or your resident discuss trust explicitly?

Did you or your resident discuss supervision explicitly?

Did you try to ask for a different level of trust or supervision?

How aware was the senior resident that what they were doing conveyed a lack of trust in you?

Why do you think this senior did this?

In general, how was your working relationship with this resident?

How long had you worked with this resident?

What do you think were the consequences for you of this resident not trusting you enough?

How did this affect your learning?

How did this affect the clinical care you provided?

Did this affect the team?

Next, I’d like to ask you to think of a time when you felt your resident supervisor trusted you too much.

What was the situation? Could you describe the clinical setting? How much supervision did you receive from your senior resident?

probe if not already mentioned:

*Acuity of the patient*

*Your familiarity w/ patient, length of time in care*

*Time of day (was it a nightshift/weekend?)*

*Overall ward census*

*Team “mood”*

What specifically made you feel that your resident trusted you too much?

Did you or your resident discuss trust explicitly?

Did you or your resident discuss supervision explicitly?

Did you try to ask for a different level of trust or supervision?

How aware was the senior resident that what they were doing conveyed a lot of trust in you?

Why do you think this senior did this?

In general, how was your working relationship with this resident?

How long had you worked with this resident?

What do you think were the consequences for you of this resident trusting you too much?

How did this affect your learning?

How did this affect the clinical care you provided?

Did this affect the team?

Last, I will ask you to think of a time when you felt you were trusted by your resident just the “right amount.”

What was the situation? Could you describe the clinical setting? How much supervision did you receive from your senior resident?

probe if not already mentioned:

*Acuity of the patient*

*Your familiarity w/ patient, length of time in care*

*Time of day (was it a nightshift/weekend?)*

*Overall ward census*

*Team “mood”*

What specifically made you feel that your resident trusted you the “right amount?”

Did you or your resident discuss trust explicitly?

Did you or your resident discuss supervision explicitly?

What was your involvement in determining these levels of trust and supervision?

How aware was the senior resident that what they were doing conveyed trust in you?

Why do you think this senior did this?

In general, how was your working relationship with this resident?

If you had to give an adjective to describe the management style of this senior resident, what would it be?

How did the relationship develop to be effective?

How long had you worked with this resident?

What do you think were the consequences for you of this resident trusting you the “right amount?”

How did this affect your learning?

How did this affect the clinical care you provided?

Did this affect the team?

Is there anything else you would like to add about residents’ trust in interns?

How often do you think about trust?

How often does the topic of trust come up in your conversation w/ supervisors?

Are there things interns can do so that their senior residents trust them more?

Thank you for participating in the interview.

1. i.e. PGY-1 resident [↑](#footnote-ref-1)
