# Supplementary material for: How supervisor trust affects early residents’ learning and patient care: A qualitative study
Source: Perspect Med Educ. 2021 Jul 23;10(6):327–33. doi: 10.1007/s40037-021-00674-9 (PMC8633204; doi:10.1007/s40037-021-00674-9)
Supplement: Supplementary file 2 — Table S1 Factors used by trainees to recognize different levels of supervisor trust. Four factors described how trainees recognized different levels of supervisor trust: supervisor—the amount and type of support and availability provided to trainees by their supervisors; task—the acuity, complexity, quantity, sequencing, and risk associated with clinical activities; relationship—interpersonal dynamics, values, concordance, communications, and amount of contact; and context—workplace culture and environment, systems issues, and workload. (Interviewees referred to PGY‑1 residents as “interns”, and to supervising PGY‑3 residents as “seniors”. Numbers in parentheses are participant ID numbers) [file 40037_2021_674_MOESM2_ESM.docx]

**Table S1** Factors used by trainees to recognize different levels of supervisor trust. Four factors described how trainees recognized different levels of supervisor trust: *supervisor* - the amount and type of support and availability provided to trainees by their supervisors; *task* - the acuity, complexity, quantity, sequencing, and risk associated with clinical activities; *relationship* - interpersonal dynamics, values, concordance, communications, and amount of contact; and *context* - workplace culture and environment, systems issues, and workload. (Interviewees referred to PGY-1 residents as “interns,” and to supervising PGY-3 residents as “seniors.” Numbers in parentheses are participant ID numbers)

| Factor | Optimal Trust | Under-Trust | Over-Trust |
| --- | --- | --- | --- |
| SUPERVISOR -  Supervisor Support | Balanced support, assessment of trainee, and teaching/guidance  “When the senior [let me] speak for the team and the patient … that conveys that the [senior] trusts [me] to do right by the team and the family as a primary physician.” (6) | Excessive support, inadequate evaluation of trainee’s skills, and redundant teaching  “She would also do [the exams] at the same time. It felt really redundant and like she really didn't have any trust in either of us.” (2) | Inadequate support, evaluation of trainee’s skills, and availability  “Sometimes I wouldn’t know entirely what to do with the data and I wanted help and … she wasn’t able to give me that.” (13) |
| TASK -  Acuity and Complexity | Clear patient care responsibilities, roles promoting critical thinking and decision-making, opportunities for growth, acuity matching competence  “I was the one making decisions as far as workups and plans and … she was going to do parts of the management [that were] least educational.” (13) | Responsibilities limited, exclusion from decision-making; often low patient acuity or quantity of tasks  “That's another thing that contrasted, the [senior] residents did end up putting orders in without telling me. Then, I didn't know about them. So, that undermined my trust. Also, undermined my learning.” (10) | Responsibilities exceeding competence; often high patient acuity and high quantity of tasks  “To the senior probably, they have done that order set a hundred times and didn't think there was anything ... I think it was an oversight.” (4) |
| RELATION-  SHIP -  Working Relationship | Mutual trust, valued team membership, open/honest communications, validation, shared expectations, mutual advocacy, bidirectional feedback  “I think … [it] is important to understand that it’s a team; to create a team environment and not just a hierarchical environment.” (14) | Dictatorial and hierarchical team structure, one-way communication from supervisor to trainee, trainee excluded from team  “It feels more like ... this monarchy where one person is doing everything and everybody else is there for no reason.” (2) | Lack of teamwork, communication, expectations, or shared values  “I realized how confusing our communication was. … I think it made me not trust the senior as much.” (12) |
| CONTEXT -  Culture and Logistics | Workplace supporting time/space for trainee autonomy, encouragement for independent thinking, and adequate staffing for teaching  “This senior, I probably worked with a little bit more, so I think through that experience ... they could tell that I was somewhat competent ... able to take on some more of the medical decision making." (5) | Workplace barriers to participation in critical thinking, lack of time/space for autonomy, and redundancy in workforce  “The teaching was at a level that would've been much more appropriate at the very beginning of the year.” (7) | Workplace barriers to asking for and receiving help (patient census, insufficient staffing, time of day)  “Over half the time I got autonomy because it was busy. It’s understandable … your senior might forget to gauge … how much experience an intern has had in the past.” (3) |
