# Supplementary material for: How supervisor trust affects early residents’ learning and patient care: A qualitative study
Source: Perspect Med Educ. 2021 Jul 23;10(6):327–33. doi: 10.1007/s40037-021-00674-9 (PMC8633204; doi:10.1007/s40037-021-00674-9)
Supplement: Supplementary file 3 — Perceived effects on the trainee from optimal, under- and over-trust. Effects were categorized into three codes which, when separated by trust level, yielded nine themes. Interviewees referred to PGY‑1 residents as “interns“, and to supervising PGY‑3 residents as “seniors.”. Numbers in parentheses are participant ID numbers [file 40037_2021_674_MOESM3_ESM.docx]

**Table S2** Perceived effects on the trainee from optimal, under- and over-trust. Effects were categorized into three codes which, when separated by trust level, yielded nine themes. Interviewees

referred to PGY‑1 residents as “interns,” and to supervising PGY‑3 residents as “seniors.” Numbers in parentheses are participant ID numbers.

| Trainee effects | Optimal trust | Under-trust | Over-trust |
| --- | --- | --- | --- |
| Learning experiences | Most useful learning experience because trainee is appropriately autonomous, and guided to learn  “I liked that she let me ask, I like that she let me tell the story, ask what I wanted to do, and then tell me when I told her ‘no I don't actually know what to do,’ and then I liked that she said ‘do you feel comfortable with this plan?’ I felt like I could say ‘yes’ or ‘no’ truly.” (8) | Learning was restricted, trainee not allowed to think independently  "The [senior's] way of doing things was to tell you what you had to do that day... like I had to be told to make a phone call. I just felt like I couldn't be trusted to do any small task.” (16) | Variable learning experiences; non-guided  “In terms of learning, there's pros and cons. … The pro is that it really pushes you to decide something and act on it, … but the con is that you feel like you're learning in the dark.” (9) |
| Attitudes and self-confidence | Empowered, valued, less vulnerable  “I felt that I learned more. I felt that I grew more... I grew in my confidence, but not over-confident because I had someone to talk that through and learn when I was in fact thinking things incorrectly or out of inexperience. Those interactions led to more opportunities to do more, which ... is better. You do more things and people get better care.” (1) | Belittled, worthless, undermined, low confidence  "If someone acts like they don't trust you, the assumption is 'Oh, I must not be good enough.’” (11) | If trainee successful, then increased confidence.  If not, then doubt and fear  “When someone's trusting you too much you feel uncomfortable. There's a difference between someone making you push your boundaries and you saying, ‘Okay, I feel a little uncomfortable, but I think I could also probably do it.’" (19) |
| Identity and role | Increased patient ownership and opportunities for participation  “[The senior resident] serve[s] as somebody that answers questions. Trusting us enough to sit back and allow us to decide when we felt like we needed help as opposed to really interjecting into our way [of] taking care of patients.” (2) | Excluded from team, lack of ownership over patient and patient care  "I wasn't given a role. The expectation was that the senior would take care of everything ... so you really didn't have a sense of ownership of any of the patients or a clear role or clear expectations.” (11) | Excessive delegation created potential for patient harm; trainee may feel lost but also increased responsibility  "Obviously [the senior] had to have a lot of trust in order to not lay those guidelines out. Some interns try to do a lot more on their own than is probably safe or expected because they don't know what the expectation is." (17) |
